# Supplementary material for: Systematic review and meta-analysis of 50 years of coral disease research visualized through the scope of network theory
Source: PeerJ. 2019 Jun 4;7:e7041. doi: 10.7717/peerj.7041 (PMC6555395; doi:10.7717/peerj.7041)
Supplement: Supplemental Information 1 [file peerj-07-7041-s001.pdf]

Systematic review of 50 years of coral disease research visualized through the scope of network theory

Luis M. Montilla, Alfredo Ascanio-Moreno, Alejandra Verde, and Aldo Croquer.

Rationale for conducting the meta-analysis

Coral diseases are an important factor of coral loss around the world, especially when combined with environmental stressful conditions. Research around these topics have been developing since 50 years ago, and there have been several narrative reviews compiling the status and progress of the field from the perspective of multiple senior researchers. Quantitative approaches providing more objective details about the whole corpus of research are not yet available, and other approximations deal with very specific issues, for example, what has been the most prevailing methods used for coral disease research? (Work et al, 2014), or the status of research on multiple stressors (including coral diseases) in coral reefs (Ban et al. 2014).

Contribution that the meta-analysis makes to knowledge in light of previously published related reports, including other meta-analyses and systematic reviews

Our work complements the aforementioned works, providing a perspective on the topics (coral diseases, coral hosts, sampled regions, and research questions) that have concentrated most of the effort in coral disease research during the last 50 years, and concomitantly, this also provides a basis for the identification of research gaps that can be explored in the future.
